# Pathways to psychiatric care in urban north China: a general hospital based study

**DOI:** 10.1186/1752-4458-7-22

**Published:** 2013-09-10

**Authors:** Weijun Zhang, Xuemei Li, Yan Lin, Xiulan Zhang, Zhiyong Qu, Xiaohua Wang, Huiwen Xu, Alvina Jiao, Mengqi Guo, Yurong Zhang, Yafang Li, Donghua Tian

**Affiliations:** 1 School of Social Development and Public policy, China Institute of Health, Beijing Normal University, 19, Xinjiekou Wai Street, Beijing 100875, China; 2 Clinics of Cadre, Department of Outpatient, General Hospital of the People's Liberation Army, Beijing 100853, China; 3 Woodrow Wilson School of Public and International Affairs, Princeton University, Princeton, NJ 08544-1013, USA

**Keywords:** Help-seeking pathways, Psychiatric care, Patients with mental illness, Mental health professional, Urban north China

## Abstract

**Background:**

Pathway studies highlight the help-seeking behaviors of patients with physical and mental illnesses. A number of studies in this field have been completed in various parts of the world. The purpose of this study is to explore the characteristics of the help-seeking pathways of patients with mental illness from urban north China at Mental Health Professional (MHP).

**Methods:**

The pathway diagrams, which accounted for more than five percent of patients, were documented for 441 subjects using the translated version of the World Health Organization (WHO) pathway encounter form. The patterns and durations of care-seeking were analyzed in different diagnostic groups. The χ^2^-test and the Mann-Whitney U test were employed, as needed.

**Results:**

Respondents visited the MHP through a variety of pathways. Approximately three-quarters of the patients took an indirect pathway (74.8% vs 25.2%, χ^2^ = 108.8, *p* < 0.0001), and on average, each patient consulted 3.4 caregivers. The vast majority of patients first visited local tertiary general hospitals (56.4% *vs* 4.1%, χ^2^ = 138.3, *p* < 0.0001) or local secondary general hospitals (24.8% *vs* 4.1%, χ^2^ = 40.96, *p* < 0.0001). However, only 9.6% of patients were diagnosed with mental disorders for the patients who first visited non-psychiatric hospitals. Of the patients who first contacted with psychiatry hospital, 55.6% received a professional diagnosis and finally reached the MHP because of the poor treatment or high-cost medical care.

**Conclusions:**

The majority of patients seek other pathways than to go to MHP directly and this may be due to stigma, and/or lack of knowledge. The study gives emphasis on the importance of improving skills and knowledge that will facilitate the recognition of psychiatric disorders in the community health centers, the general hospitals system and by private practitioners. The pathway described by this study may be helpful while preparing mental health programs in the future.

## Introduction

An understanding of the help-seeking behaviors of patients with mental illness is crucial to the effective planning of psychiatric services across the country [1]. An exact knowledge about the help-seeking pathways of patients is pivotal in providing early interventions and thereby in supplying specialized and focused health care [2]. The pathways toward mental illness care are diverse and dependent on socio-cultural and economic factors, including the conventions governing referral, the availability/accessibility of mental health services, and the relationship between mental health services and the rest of the disciplines [3,4]. Currently, China is undergoing a rapid economic and social transformation accompanied by dynamic changes in all aspects of lifestyle, including mental health services [5]. Mental health has gradually become a population-wide health problem. A recent study estimated that roughly 173 million Chinese, or nearly one in five adults, suffer from a mental disorder as defined by the Diagnostic and Statistical Manual (DSM)-IV [6]. Most of these patients do not seek professional help and are left to their own devices [7]. Apart from the increasing mental health burden, China, like many developing countries, is facing a severe shortage of skilled mental health professionals and bed provisions [8]. Before 2011, there were 20,914 licensed psychiatrists and assistant psychiatric practitioners (1.55/100,000 population) and 38,907 licensed psychiatric nurses (2.89/100,000 population) [9], far below the global average of 4.15 psychiatrists and 12.97 psychiatric nurses per 100,000 population [10,11], and also farther below the Africa average of 10.5 psychiatrists and 34.5 psychiatric nurses and the South East Asia average of 4.54 psychiatrists and 9.3 psychiatric nurses, per 100,000 population [10]. Only 1% of the one million total medical graduates in China took up a career as a psychiatrist [12]. Of 690 psychiatric hospitals, most (57.1%) still concentrated in metropolitan and urban areas [9]. Among them, 475 (68.8%) had 100 or more beds and 114 (16.5%) had 500 or more beds. The total number of psychiatric beds was 225,641, i.e., 1.58/10,000 population [9]; this is also significantly lower than the global average of 4.36/10,000 population of psychiatric beds [10]. In addition to the shortage of beds and skilled mental health professionals, there is a concern about the variable standard of care provided. At present, there is no accreditation mechanism in place to assess the level of competence of psychiatrists in China, leading to wide variation in the quality of care delivered by psychiatrist and mental health professionals in different areas [8,13]. To remedy this issue the Ministry of Health, now re-named the National Health and Family Planning Commission of the People’s Republic of China, has started to develop a three-year clinical training program for specialist psychiatrists [8]. A promising development was the initiation of a national community-based service delivery model in psychiatry (the 686 project) in 2004 [14]. This project integrated the resources of psychiatric hospitals and existing community health systems, with the aim of training a core group of mental health professionals in case management and the use of individual service plans to enable them to deliver training programs and establish community-based services [14,15]. However, the project is primarily for those with severe mental illness, rather than the patients at the hospital outpatient department. Unlike other countries there is no discrimination against coverage for mental health services. China does not have a national health insurance system in which mental health services are available to all [8], the coverage of insurance is limited for inpatient services, but it is no more limited for mental health services than for other types of services, many types of mental disorders are not covered by these insurance programs in many areas, although the current insurance system covers the vast majority of the population in China. Some patients with severe mental disorders remain untreated, especially in economically underdeveloped areas, possibly due to the economic reasons [16]. National programs in China are under enormous pressure to provide mental health services necessary to a large population [6], and the development of a successful service is also a challenge for concerned authorities.

Epidemiological studies in China have reported that among individuals with a diagnosable mental illness, 24% were moderately or severely disabled by their illness, 8% had sought professional help, and 5% had seen a mental health professional [6]. Among those patients who had received professional help, 41% had only been treated by non-mental health professionals, mainly physicians who practice Western medicine or traditional Chinese medicine [6]; this also indicated the necessity to explore the characteristics of help-seeking pathways of patients with mental illness to improve the mental health services system in China.

Pathway studies highlight the help-seeking behaviors of patients with physical and mental illnesses. A number of studies in this field have been completed in various parts of the world [17-20], and also including Japan, Bangladesh, and India. However, there have been few reports from China. Therefore, this study was planned to investigate the characteristics of help-seeking pathways adopted by patients with mental illness from urban regions, as well as to analyze the durations and previous diagnoses. The study examined biomedical care providers grouped into five major categories according to the present health system in urban China, including Secondary General Hospital (SGHs), Tertiary General Hospitals (TGHs), the hospitals of Traditional Chinese Medicine (TCM), psychiatric hospitals, Community Health Centers (CHCs), and the direct pathways to MHP.

## Methods

### Study setting

In China, the urban essential health care system is organized into three tiers: community health service stations, community health centers (CHCs), which provide preventive services, medical treatment and health care, and district hospitals (equal to secondary general hospitals), which constitute the final tier of three-tiered system and provide professional care for serious cases. To ensure a higher quality of medical care, the government has also established many large and comprehensive hospitals (tertiary general hospitals) in different regions [21]. In addition, there are many other types of health care, including the hospitals of traditional Chinese medicine (TCM), psychiatric hospitals and private clinics. Meanwhile, an integrated mental health system, a three-tier model centered on provincial capitals and major cities, has been gradually developed throughout China since the early 1990s [22]. Although this three-tier system only exist in a few big cities, most cities in China do not yet have any community mental health workers of any kind. Mental health institutes in the provinces or major cities constitute the top tier of the system and are responsible for enacting management guidelines and providing guidance and support to all mental health services in the area. The middle tier (town or district level) comprises mental health institutes in medium-size cities (population of several millions) or psychiatric hospital in towns and rural areas. The bottom tier based in the community offices and village employs mental health field with responsibilities similar to community psychiatric nurses (albeit without formal qualifications) in the West [8]. Mental health worker provide regular support and contact with patients and their caregiver, and deliver some forms of rehabilitation [8]. Over time, the Chinese government has successfully implemented this three-tier mental health system in most cities in China [23]. Such a structure is crucial in the delivery of interventions to the local population. However, one major obstacle in the delivery of mental health services is the fee-for-service system used by most hospitals since the introduction of an open market economy. The overwhelming majority of hospitals have become financially self-sufficient and have adopted a marketing strategy of prioritizing services for people with secure medical insurance or stable employment [8]. As most patients with severe mental illness are likely to be unemployed and financially depend on their family, many hospitals are reluctant to provide mental health services with a limited return on investment [13]. As a result, the mental health care system in China lacks equality of access [8], it is accessible only to the insured and/or wealthy urban populations [24]. Although the central government has increased funding for some mental health care initiatives, this gesture remains a far cry from the long-term commitment necessary to ensure adequate treatment for persons with mental illness.

### Sample

In Beijing, there are 19 psychiatric hospitals with 5,443 psychiatric beds (5 provincial hospitals, 11 prefectural hospitals, and 3 county hospitals), and 6 psychiatric outpatient departments with 913 psychiatric beds in general hospitals [25]. This hospital-based study was performed from October 2010 to September 2011 in the outpatient department of neurology at the PLA general hospital, which is one of the best medical centers in China and integrates medical care, health care, education and research across all disciplines with an excellent medical care environment. In addition to providing healthcare services to the troops stationed in Beijing, PLA general hospital is also open to civilian patients from all over the country. It also has a reputed mental health service, with 11 psychiatrists and 55 physicians with psychiatric qualifications, and the outpatient number is approximately 100 per day. Hence, it was regarded as the MHP in this study. The pathways to care were defined as the path a psychiatric patient travels during his referral process to a MHP. Accounting for feasibility issues in the participating mental health care system and using previous experience with the pathways method, which included 50 subjects per center, a sample size of 441 was considered sufficient for a meaningful analysis [1,3,19]. Using the convenience sampling method, all of those who were newly referred to MHP and agreed to participate in this research were interviewed until the target 441 participants were recruited. Newly referred patients were defined as general population with mental disorders who first visited the PLA general hospital and had not sought care from mental health services during the previous year. In addition, the patients who were transferred from other departments within PLA general hospital were excluded from the sample, as the purpose of this study is to investigate the referral system among the different institutions of the three-tier health system in China. The research protocol was approved by the institutional review board of Beijing Normal University (BNU) and the research oversight committee of PLA general hospital. For subjects who were unable to answer the questions due to a diagnosis of severe mental illness, family members or relatives who had accompanied them to hospital were interviewed; for respondents under 18 years old, their parents were interviewed. The informed written consent of the participants was obtained prior to each interview.

### Data collection

This study adopted the methodology of the WHO pathway study [3] and the multi-center pathway study conducted in Eastern Europe [19]. Each participant was interviewed using a semi-structured questionnaire that was prepared based on the encounter form developed in the WHO collaborative study. Specifically, we translated and retranslated the encounter form to and from Chinese (Mandarin). We then compared the original and retranslated English versions, consulting with linguistics experts as well as qualified persons working in the mental health field to revise the Mandarin translation as necessary. Finally, the questionnaire was used to gather information on the socio-demographic characteristics of the participants and their sources of care before reaching the MHP.

For further treatment, the confirmed diagnoses were independently made by at least three psychiatrists according to the latest version of the *Chinese Classification of Mental Disorders (CCMD-3)*[26]*,* which was published by the Chinese Psychiatric Association in 2001. Its descriptive definitions and diagnostic criteria were based on the clinical descriptions and diagnostic guidelines of the *WHO International Classification of Disease (ICD-10)* and the *Diagnostic and Statistical Manual of Mental Disorders (DSM-IV)*, respectively [26]. Six psychiatrists in charge administered the questionnaire and the interview, which took 10-15 minutes per patient. All of the psychiatrists were trained for 36 hours by the leader of research group, and an instruction and coding manual was supplied to each psychiatrist who took part in this study.

### Analysis of data

The routes taken by the participants were compiled into a pathway diagram that was marked with proportions. The time intervals between the onset of the problem, the first time seeking care and arrival at the MHP were analyzed among diagnostic groups. Categorical data were analyzed using the χ^2^-test. Continuous variables (such as the duration of the problem) were highly skewed; therefore, average values are presented as medians, and the Mann-Whitney U test was employed as needed.

## Results

### Sample characteristics

A total of 441 participants were included in this study, and the average age of the participants was 46.17 years (SD 14.9), with a range of 13-86 years. For the 28 subjects who were unable to answer the questions due to severe mental illness, immediate family members (23/441) or other relatives (5/441) were interviewed. For the 10 subjects who were under 18 years old (10/441), their parents were interviewed. Of 441 participants, females comprised nearly two-thirds of the sample (74.8% vs. 25.2%, χ^2^ = 108.8, *p* < 0.0001). The participants in this sample came from Beijing (22.6%), Tianjin (20.9%), Hebei (20.0%), Shanxi (17.2%), and Inner Mongolia (19.3%). Of all subjects, 80.0% were married and living with their partners. In addition, 30.1% graduated from university and above while, 27.9% attended school up to a primary education level (junior middle school level and below) (Table 1). It is noteworthy that the number of retired subjects is 33.1%, which represents the highest ratio out of all subjects (Table 1).

**Table 1:** Socio-demographic data

**Number of subjects**	**441**
Age (average) (SD)	46.17 (14.9)
Sex (Percent)	
Male	160 (36.3)
Female	281 (63.7)
Marital status (Percent)	
Married	353 (80.0)
Widowed	22 (5.0)
Single	66 (15.0)
Occupation (Percent)	
Government/Enterprise/Institutions administrators	74 (16.8)
Professional and Technical personnel	54 (12.2)
Clerk	40 (9.1)
Business/Service personnel	14 (3.2)
Self-employed workers or merchants	35 (7.9)
Individual industrialist and businessman	5 (1.1)
Retired	146 (33.1)
Unemployed	44 (10.0)
Student	25 (5.7)
Others	4 (0.9)
Educational level (Percent)	
Junior middle school and blew	123 (27.9)
Technical (secondary) school/High school	101 (22.9)
Junior college	84 (19.0)
University	109 (24.7)
Graduate and above	24 (5.4)
Region (Percent)	
Beijing	100 (22.6)
Tianjin	92 (20.9)
Hebei	88 (20.0)
Shanxi	76 (17.2)
Inner Mongolia	85 (19.3)

### Presenting features and diagnoses in MHP

At the MHP, the most frequent diagnoses for all patients were neurosis (F40-F49, excluding anxiety disorders and generalized anxiety disorders, somatoform disorders and tension headache in this study), depression (F32), somatoform disorders (F20) and anxiety (F41 and F41.1), which accounted for 23.1%, 18.6%, 12.2%, and 7.9% of patients, respectively. The patients with organic mental disorders, including mental disorders due to Alzheimer’s disease and mental disorders due to vascular disease, accounted for 4.2% of the sample, and essential prescriptions were provided to them to control the development of these conditions. In addition, 17.5% of all participants had symptoms of depression and anxiety, but the symptoms were milder and not diagnosed as depression or anxiety by the psychiatrists according to the diagnostic standards (Table 2). These respondents were not excluded from the study in view of their help-seeking behavior for mental health problems, and psychological services and essential prescriptions were provided to control the development of these conditions by the psychiatrists. Altogether, 54 patients (12.24%) reported that they had a previous family psychiatric history, but higher rates were found in the depression group, the insomnia group, and the anxiety group (Table 2). However, the distribution above should not be interpreted as the difference in the prevalence of mental illness. Comparatively, depression (χ^2^ = 9.56, *p* = 0.002), neurosis (χ^2^ = 7.69, *p* = 0.006), neurasthenia (χ^2^ = 6.25, *p* = 0.012), and organic mental disorders (χ^2^ = 13.33, *p* < 0.0001) were more prevalent in females than in males (Table 3); this disparity may also reflect the results of the nationwide epidemiological study [6]. In addition, statistical analysis was not performed on other groups with sample sizes less than 10, including the schizophrenia group and the group with hysterical psychological disorders.

**Table 2:** Previous history and current diagnosis

**CCMD-3 diagnostic group**		**Previous psychiatric history**
	**n (%)**	**n (%)**
Neurosis^a^ (F40-F49)	102 (23.1)	11 (20.3)
Depression (F32.0)	82 (18.6)	11 (20.3)
Somatoform disorders (F45)	54 (12.2)	3 (5.6)
Anxiety^b^ (F41+ F41.1)	35 (7.9)	6 (11.1)
Neurasthenia (F48.0)	16 (3.6)	1 (1.9)
Organic mental disorders^c^ (F00 + F01)	30 (4.2)	2 (3.7)
Insomnia (F51.0)	26 (5.9)	7 (13.0)
Bipolar disorders (F31)	13 (3.0)	1 (1.9)
Schizophrenia (F20)	5 (1.1)	2 (3.7)
Hysterical psychological disorders (F44.8)	1 (0.2)	0
Status of depression and anxiety^d^	77 (17.5)	10 (18.5)
Total	441 (100)	54 (100)

^a^ Neurosis doesn't cover Anxiety disorder, Generalized anxiety disorder, and Somatoform disorders;

^b^ Anxiety includes Anxiety disorder and Generalized anxiety disorder;

^c^ Organic mental disorders mainly include Mental disorders due to Alzheimer’s disease (F00), Mental disorders due to Vascular disease, Epilepsy, and involutional syndrome;

^d^ Participants have symptoms of depression and anxiety, but the symptoms were milder and were not diagnosed as depression or anxiety eventually by the diagnostic standard.

**Table 3:** The distribution of each diagnostic feature between males and females

**CCMD-3 diagnostic group**	**Male**	**Female**	**χ**^**2**^	**df**	***P***
	**n (%)**	**n (%)**			
Depression (F32.0)	27 (32.9)	55 (67.1)	9.56	1	0.002
Neurosis (F40-F49)	37 (36.3)	65 (63.7)	7.69	1	0.006
Anxiety (F41+ F41.1)	17 (48.6)	18 (51.4)	0 03	1	0.866
Somatoform disorders (F45)	23 (42.6)	31 (57.4)	1 185	1	0.276
Neurasthenia (F48.0)	3 (18.8)	13 (81.3)	6.25	1	0.012
Organic mental disorders (F00 + F01)	5 (16.7)	25 (83.3)	13.33	1	<0.001
Insomnia (F51.0)	8 (30.8)	18 (69.2)	3846	1	0.05
Schizophrenia^a^ (F20)	4 (80.0)	1 (25.0)			
Bipolar disorders (F31)	4 (30.8)	9 (69.2)	1.923	1	0.166
Status of depression and anxiety	32 (41.6)	45 (58.4)	2.20	1	0.138
Hysterical psychological disorders^a^ (F44.8)	0	1 (100)			
Total	160	281			

Note: ^a^indicated that the statistical analysis was not performed because the sample size is too small (less than 10 patients).

### Pathways to care

**Figure 1:**
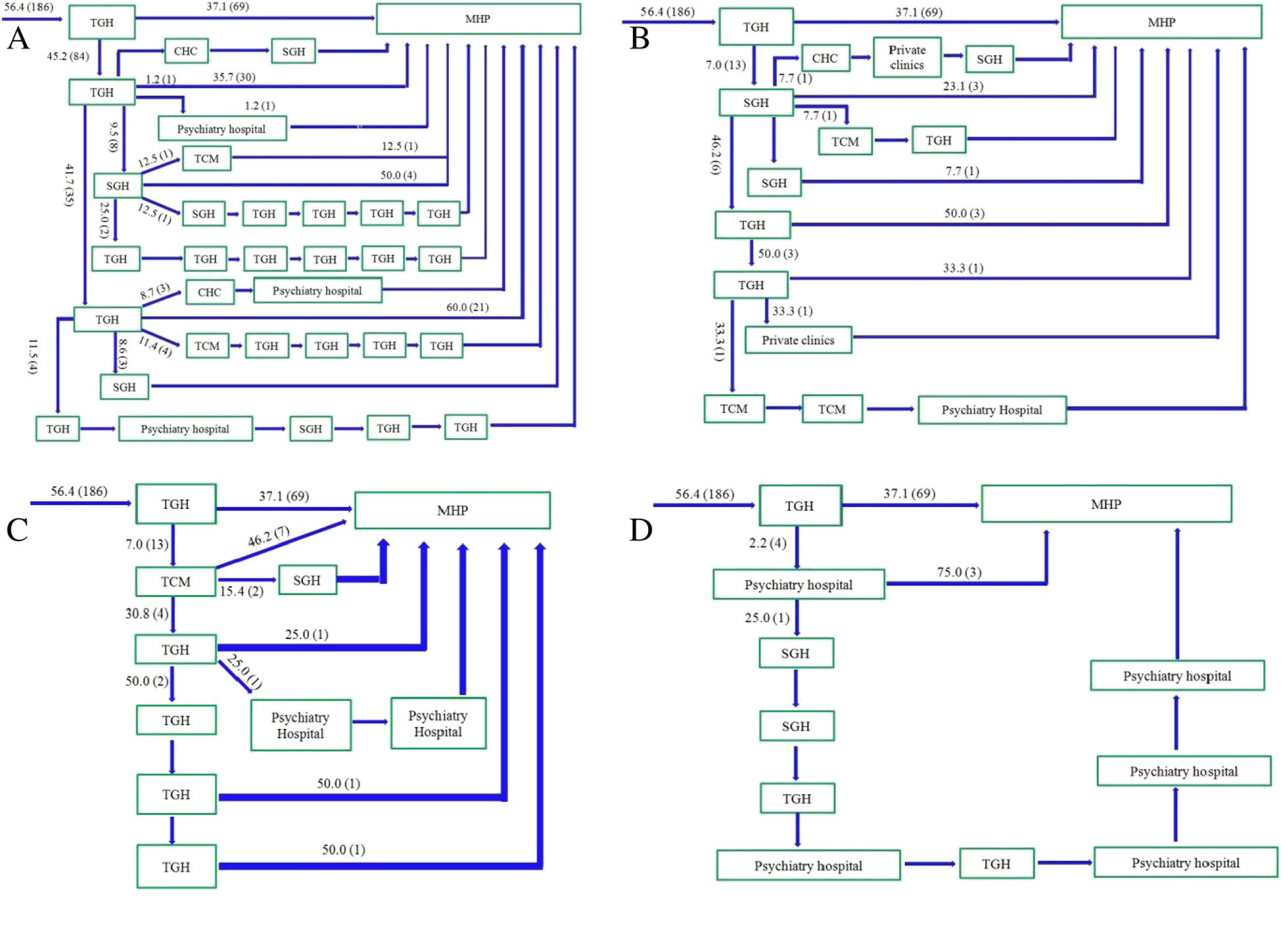
**The psychiatric hospitals pathway.** Note: MHP—Mental Health Professional; SGHs—the secondary general hospitals; TGHs—the tertiary general hospitals; TCMs—the hospitals of traditional Chinese medicine; CHCs—Community Health Centers. Among the patients who took the indirect pathway, 18 patients (5.5%) first visited the local psychiatric hospital. Afterwards, most patients also visited the SGH and TGH and eventually arrived at the MHP.

**Figure 2:**
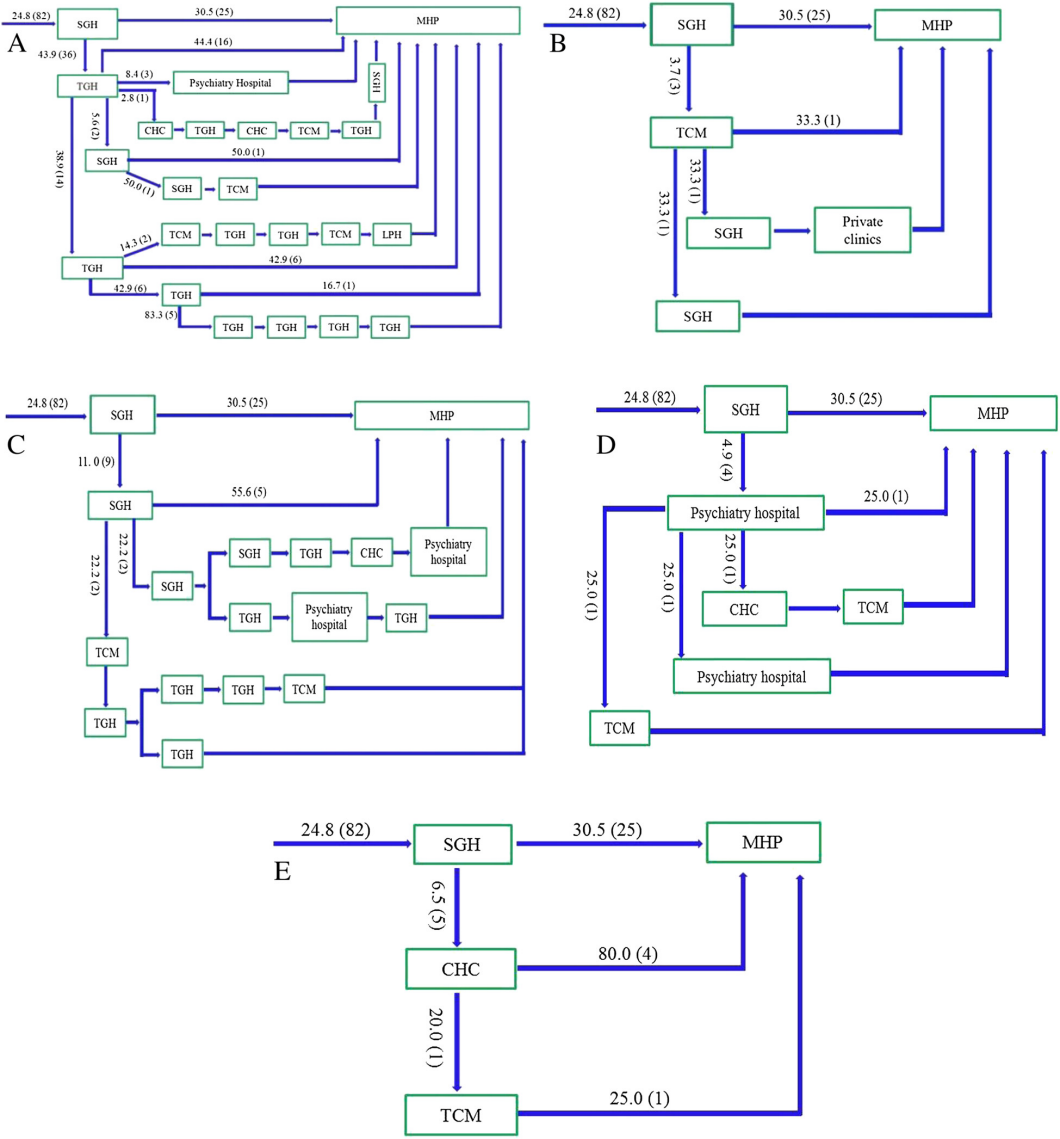
**The tertiary general hospitals (TGHs) pathway.** Note: MHP—Mental Health Professional; SGHs—the secondary general hospitals; TGHs—the tertiary general hospitals; TCMs—the hospitals of traditional Chinese medicine; CHCs— Community Health Centers. Among the patients who took the indirect pathway, 186 patients (56.4%) first visited the TGHs. Afterwards, most patients also went to the SGH and TGH and eventually arrived at the MHP. **A**: TGH-TGH pathway (45.2%); **B**: TGH-SGH pathway (7.0%); **C**: TGH-TCM pathway (7.0%); **D**: TGH-Psychiatric hospital (2.2%).

**Figure 3:**
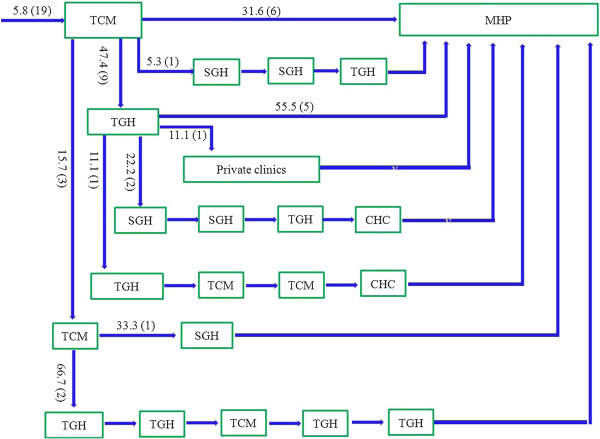
**The secondary general hospitals (SGHs) pathway.** Note: MHP—Mental Health Professional; SGHs—the secondary general hospitals; TGHs—the tertiary general hospitals; TCMs—the hospitals of traditional Chinese medicine; CHCs— Community Health Centers. Among the patients who took the indirect pathway, 82 patients (24.8%) first visited the SGHs. Afterwards, most patients also went to the SGH and TGH and eventually arrived at the MHP. **A**: SGH-TGH pathway (43.9%); **B**: SGH-TCM pathway (3.7%); **C**: SGH-SGH pathway (11.9%); **D**: SGH-Psychiatric hospital (4.9%); **E**: SGH-CHC pathway (6.5%).

**Figure 4:**
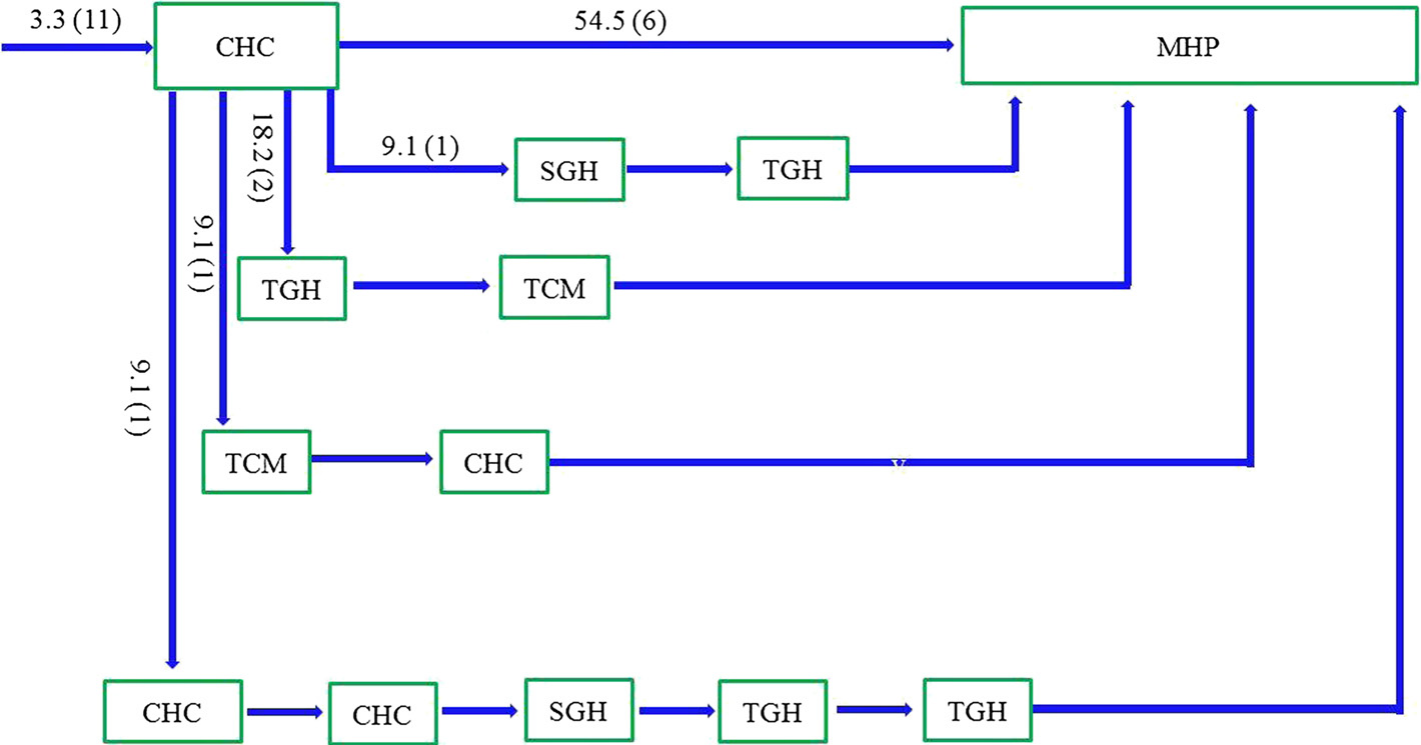
**The hospitals of traditional Chinese medicine (TCMs) pathway.** Note: MHP—Mental Health Professional; SGHs—the secondary general hospitals; TGHs—the tertiary general hospitals; TCMs—the hospitals of traditional Chinese medicine; CHCs— Community Health Centers. Among the patients who took the indirect pathway, 19 patients (5.8%) first visited the hospital of traditional Chinese medicine. Then, most patients also went to the TCM (15.7%), TGH (47.4%) and MHP (31.6%), only a few visited other hospitals. Afterwards, all the patients eventually arrived at the MHP through various pathways.

**Figure 5:**
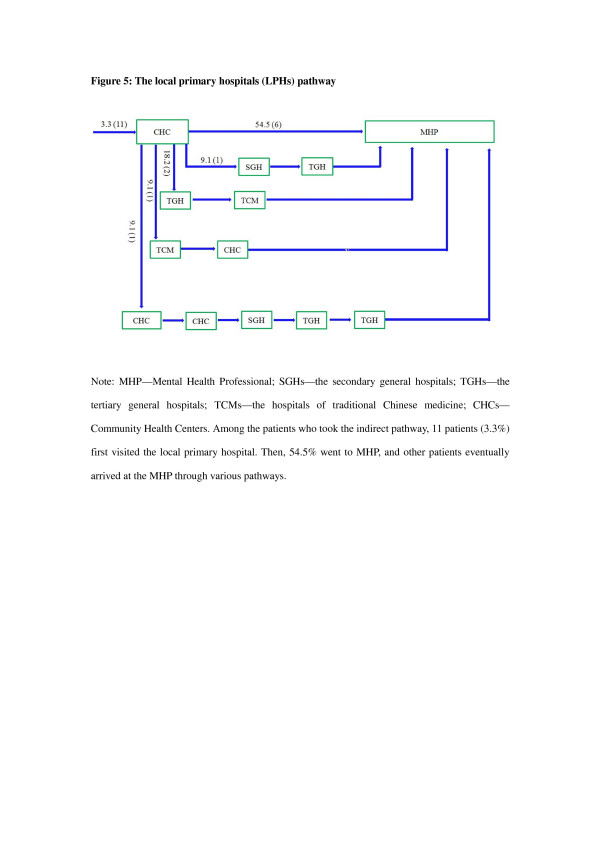
**The local primary hospitals (CHCs) pathway.** Note: MHP—Mental Health Professional; SGHs—the secondary general hospitals; TGHs—the tertiary general hospitals; TCMs—the hospitals of traditional Chinese medicine; CHCs— Community Health Centers. Among the patients who took the indirect pathway, 11 patients (3.3%) first visited the local primary hospital. Then, 54.5% went to MHP, and other patients eventually arrived at the MHP through various pathways.

Approximately three-quarters of patients took an indirect pathway to care (74.8% vs 25.2%, χ^2^ = 108.8, p < 0.0001). Only 5.5% of patients made their first contact with a psychiatric service (Figure 1), while the vast majority of patients first visited local tertiary general hospitals (56.4% *vs* 5.5%, χ^2^ = 138.3, *p* < 0.0001, Figure 2) or local secondary general hospitals (24.8% *vs* 5.5%, χ^2^ = 40.96, *p* < 0.0001, Figure 3). In addition, there were no significant differences in other care centers, including the hospitals of traditional Chinese medicine (5.8%, Figure 4), and community health centers (3.3%, Figure 5), compared to the psychiatric services. This tendency continued in later help-seeking behaviors. The direct pathway was the third most common pathway after tertiary general hospitals and secondary general hospitals pathway. However, only 9.6% of patients were diagnosed with mental disorders, 73.2% did not receive an accurate diagnosis (e.g. normal or no abnormal findings were observed etc.), 17.2% were diagnosed with other diseases, such as cardiovascular disease, digestive diseases, and so on, among the patients who first visited non-psychiatric hospitals. Of the patients who first sought help from psychiatry hospital, 55.6% received a professional diagnosis and finally reached the MHP because of the poor treatment or high-cost medical care.

### Social network for patients

On the whole, symptoms of onset were first noticed by the individuals themselves (95.7%). Furthermore, most participants (58.2%) sought consultations from local hospitals; 8.7% took some medication without consulting any doctors; 28.1% did not seek any form of help; and only a small percentage (approximately 2.8%) called the “120” emergency hotline to seek medical help as their symptoms appeared. The suggestion to first seek care most often came from the individuals themselves (80.0%), followed by family members (18.6%), relatives/friends (0.9%) and colleagues (0.2%). The vast majority (93.6%) of patients and their family members had poor knowledge of mental illness, which may be one of the reasons that patients with mental illness often diverge from multifarious care facilities. In addition, most of the patients (64.6%) went to their first caregivers alone, while a minority were accompanied by their spouse (17.2%), relatives (12.5%) or parents (5.4%).

### Durations, diagnoses and steps in the pathway

Among all patients, the median total duration from the onset of symptoms to the arrival at the MHP was 135 weeks (mean 306 weeks), with a range of 2-2684 weeks. The total duration from the onset of symptoms to reaching the MHP tended to be significantly shorter for the direct pathway than for the indirect pathway (Mann-Whitney U test, *P* = 0.01, Table 4). For the indirect pathway, the median duration from the onset of symptoms to the arrival at the initial caregiver was 9 weeks (mean 91), with a range of 2-2044 weeks. On average, each patient consulted 3.4 care givers for a median of 3 consultations and a range of 1-10 consultations. In addition, we compared the median delay from onset to meeting with the initial care giver and the median delay from the initial care giver to the MHP among the most frequently diagnosed groups (sample size > 10). The anxiety group and the organic mental disorder group each had the longest median delays from the onset to the first care giver; then followed by the insomnia group, the neurasthenia group, the bipolar disorders group, the neurosis group, the depression episode group, the status of depression or anxiety group and the somatoform disorders group (Table 5). However, the median delays from the first care giver to the MHP presented different tendencies across all patients; the bipolar disorders group topped the list with the largest median delay, followed by the neurasthenia group, the insomnia group, the status of depression or anxiety, the somatoform disorders group, the neurosis group, and the depression episode group; the organic mental disorders and anxiety groups were at the bottom of the list, with the shortest median delays (Table 5). Generally speaking, the median duration of time from the initial care giver to the MHP was longer than that from the onset to the initial care for all patients (Mann-Whitney U test *p* < 0.005) and all the disease groups. For those who followed an indirect pathway, only 12.2% of the patients were diagnosed with mental disorders, 64.1% did not receive an accurate diagnosis (e.g., normal or no abnormal findings were observed etc.), and 23.7% percent of these patients were diagnosed with other diseases, such as cardiovascular disease before they reached MHP. Professional health services, including high-quality healthcare and experienced doctors, was most common reason (30.2%), followed by recommendations from their relatives and friends (17.0%), medical insurance-designated hospitals (17.5%), proximity to home (5.7%), and other reasons (15.4%). However, a referral from their doctors was the last reason, accounting for only 1.8% of responses.

**Table 4:** The median delays from onset to MHP among the different pathways

**Diagnoses group**		**Direct pathway**	**Indirect pathway**	
**(CCMD-3)**				
	**No of patients**	**Week**	**No of**	**Week**
		**Median (mean)**	**patients**	**Median (mean)**
Depression	20	117.6 (246.4)	62	108.9 (270.1)
Neurosis	28	95.9 (180.6)	74	150.3 (350.3)
Anxiety	8	42.4.8 (72.4)	27	213.5 (325.8)
Insomnia	10	84.9 (554.6)	16	320.3 (511.0)
Somatoform disorders	6	143.8 (320.6)	48	119.8 (256.2)
Neurasthenia	4	126.4 (115.4)	12	326.8 (409.2)
Bipolar disorders	5	74.1 (73.2)	8	315.8 (438.4)
Organic mental disorders	12	564.3 (648.9)	18	213.3 (457.1)
Schizophrenia	0	0	5	217.9 (344.2)
Hysterical psychological disorders	0	0	1	26.14
Status of depression or anxiety	18	111.1 (210.8)	59	148.1 (278.9)
Total	111		330	

**Table 5:** Durations according to the main features at initial care and MHP

**Main diseases/features diagnosed by PLGA**			**First care (no. of patients)**							**Delays and steps**		
	**Total n**	**MHP n (%)**	**TGH n (%)**	**SGH n (%)**	**CHC n (%)**	**TCM n (%)**	**Community doctor n (%)**	**Private clinics n (%)**	**Psychiatric hospital n (%)**	**Onset to first carer (weeks) median (mean)**	**First care to MHP (weeks) median (mean)**	**Steps needed mean (SD)**
Depression episode	82	20 (24.4)	32 (39.0)	17 (20.7)	1 (1.0)	2 (2.4)	1 (1.2)	2 (2.4)	4 (4.9)	11 (126.6)	98.6 (187.9)	3.46 (1.84)
Neurosis	102	28 (27.5)	42 (41.2)	22 (21.6)	2 (5.3)	6 (5.9)	1 (1.0)	1 (1.0)	0	13 (109)	99.7 (204.3)	3.1 (1.48)
Anxiety	35	8 (22.9)	15 (42.9)	7 (20.0)	1 (2.9)	2 (5.8)	0	1 (2.9)	1 (2.9)	69.5 (325.5)	77.1 (229.8)	3.2 (1.00)
Insomnia	26	10 (38.5)	11 (42.3)	1 (3.8)	2 (7.6)	1 (3.8)	0	0	1 (3.8)	61 (402)	114.6 (204.5)	2.93 (1.40)
Somatoform disorders	54	6 (11.1)	29 (53.7)	12 (22.2)	1 (1.9)	4 (7.5)	0	1 (1.9)	1 (1.9)	4 (65.4)	104.4 (222)	3.5 (1.87)
Neurasthenia	16	4 (25.0)	9 (56.3)	1 (6.3)	0	2 (12.5)	0	0	0	39 (168)	168 (224)	2.4 (0.96)
Organic mental disorders	30	12 (40)	11 (36.7)	6 (20)	0	1 (3.3)	0	0	0	69.5 (325)	77.1 (229.8)	2.37 (1.33)
Bipolar disorders	13	5 (38.5)	5 (38.5)	0	1 (7.7)	0	0	0	2 (15.4)	39 (61.4)	248.5 (412.6)	3.23 (2.89)
Schizophrenia	5	0	1 (20)	0	1 (20)	0	0	1 (20)	2 (40)	42.3 (57.4)	0	1
Hysterical psychological disorders	1	0	1 (100)	0	0	0	0	0	0	70	8	3
The status of depression or anxiety	77	18 (23.4)	31 (40.3)	15 (19.5)	4 (5.2)	1 (1.3)	0	3 (3.9)	5 (6.5)	9 (79.8)	107.1 (246.4)	2.9 (1.76)

## Discussion

This is likely the first pathway to mental health professional followed by patients in Northern urban China. The study was conducted with minimal resources, and relied entirely on voluntary contributions.

### Sample

All the participants came from north urban China, which might be over-represented as a region in this sample, possibly due to location of the study center in the metropolis of Beijing. In a previous study [6], alcohol abuse, generalized anxiety, alcohol dependence, major depressive disorders and schizophrenia had a prevalence greater than 0.5%; however, no subjects were diagnosed with alcohol abuse or dependence in this study. This result is most likely connected with the objective and methods of the current study, which reflected a general-hospital-based study rather than an epidemiological survey.

### Pathways

The patients tended to consult different types of care for their physical or mental illnesses, including tertiary general hospitals, secondary general hospitals, psychiatric hospitals, the hospitals of traditional Chinese medicine, community health centers, and direct pathways, these results were similar to those from our investigation in rural region [27]. Moreover, an overwhelming majority (94.5%) of patients selected non-psychiatric resources as their care givers, and most of these patients first visited tertiary general hospitals (56.4%, Figure 2) or secondary general hospitals (24.8%, Figure 3). These results differ from our study of rural regions, in which more patients first had contact with secondary general hospitals (35.5%) than with tertiary general hospitals (32%) [27]. The complex forms of the help-seeking pathways may be related to the structure of the health care system in China, in addition to the awareness of mental disorders, the development of psychiatry, and the stigma of mental illness.

Recognition of mental disorders is poor among the general public; the overwhelming majority (93.6%) of patients and caregivers in this study had poor knowledge about mental illness. Some beliefs contend that mental illness is a result of evil spirits invading one’s body [28] or serves as a form of punishment for the wrongdoing of an individual or his ancestor. Furthermore, the development of psychiatry has been interwoven with the Chinese emphasis on social order. Persons with mental illness have always been seen as a potential source of social instability because it was feared that they could behave in an out-of-control manner [24]. These perspectives result in a strong stigma related to mental illness [29]. This stigma is not only perceived to be associated with mental illness by the Chinese community workers [30], but also contribute to the severe lack of resources dedicated to mental health care [31]. Currently, resources for mental health care are more limited than those for general medical care.

Goldberg and Huxley proposed five levels and four filters in the pathways to psychiatric care in communities [32]. The five levels include the prevalence of psychiatric disorders in the community, the prevalence of mental illness in those who consult their General Physician (GP), the prevalence of mental disorders correctly identified by the GP, the prevalence of psychiatric morbidity referred to specialist mental health services, and the prevalence of patients with mental disorders admitted to hospitals. The first filter is the health-seeking behaviors of patients, the second is the ability of the GP to diagnose a mental disorder correctly, the third is the referral to specialized mental health services, and the fourth is the decision by the specialized mental health services to admit the patient to the hospital [33]. In China, the first filter should represent illness behaviors, awareness of symptoms and self-referrals. However, the second and third filters may be more important because of the variable ability of primary health care physicians to diagnose and refer patients. These factors are gaining importance alongside the increasing availability of primary health care services in China. In order to establish community-based services, the 686 project, which integrated the resources of psychiatric hospitals and existing community health systems, was launched in 2004 [14,15], such program are now being piloted in many major cities. In addition, there is interest in developing a national accreditation system for specialist psychiatrist in China, which is similar to those currently implemented in Western countries [34].

### Social network involvement

The suggestion to seek initial help came mostly from the individuals themselves (80%) or from immediate family members (18.5%), but infrequently from relatives, friends or colleagues (1.3%). These results affirmed that the Chinese family is the base of all social support networks. It is estimated that 90% of Chinese patients with severe psychotic disorders are receiving care from family members due to a lack of residential services in the community [35]. In addition, patients in China with mental illness generally face the socially awkward situation of the absence of a social network and social support in China. Previous research has shown that individuals with severe mental illness have smaller social networks than general population [36,37]. Only families and close family friends were involved in the early stages of help-seeking amongst Chinese Canadians suffering from mental illness [38]. Another previous study has shown that patients’ social networks and social support may impact their utilization of psychiatric services [36]. The potential positive role of families should be fully realized as the mental health program is further designed in the future.

### Duration and previous diagnosis and treatment

The median duration of time from the initial care to the MHP was longer than that from the onset to the initial care in all groups but the insomnia group. These results showed that primary care, even at the local general hospital, did not act as gatekeeper or a referral proponent to psychiatric services, and there is no effective referral system in China, unlike that in Western Europe [3], Eastern Europe [19] and Africa [39,40]. In Asia, an effective referral system has been established successfully in Japan and Bangladesh [1,41]. In China, however, patients who went to non-psychiatric hospitals did not receive accurate diagnoses, professional treatment or timely referrals. For patients who first contacted with non-psychiatric hospital, only 9.6% were diagnosed with mental disorders in their first caregiver, and only 12.2% of them were diagnosed with mental disorders before they reached the MHP. China is facing a severe shortage of skilled mental health professionals, and the mental health system suffers from a severe lack of resources and low quality of care in China. Doctors working in community primary care or clinics have little or no education on the detection, diagnosis and management of mental disorders [42]. There are no departments of psychiatry in many general hospitals, and many doctors have only poor knowledge regarding mental illness. In addition, few graduates (bachelor’s degree or above) in clinical medicine received curricular education about mental health in medical school, which further contributes to the low awareness rate of mental illness. Another survey reported that most mental health practitioners had degree majors related to psychology, medicine, or education, although a substantial number had training in majors less directly related to mental health. Less than half of the sample was certificated, and nearly 40% was not currently affiliated with a professional association [5].

We have also taken note that even those patients who visited psychiatric hospitals and were diagnosed correctly during their help-seeking process finally reached the MHP because of poor treatment or high-cost medical care. This finding indicates that the shortages in treatment resources, poor quality of service, and high medical costs are still points of concern for the future. Some patients with severe mental disorders remain untreated, because insurance programs in many areas do not cover psychosis. Patients with mental disorders also expected their counselors to be highly knowledgeable, skillful, affable, experienced, talkative, and ethical [5,43], but these expectations frequently were not met. The pathways study has posed many problems, but if further progress is to be made, there must be a shift toward a more evidence-based culture and a reduction of the stigma associated with mental illness.

Over the past decade, the Chinese government has issued a series of statements emphasizing the importance of mental health [8], and high-quality studies are increasingly being funded by the government. However, transforming research results into the real-world clinical practices is a formidable problem for a country with a population of over 1.3 billion people. The 686 project, which integrated the resources of psychiatric hospitals and existing community health systems, is a national community-based service delivery model in psychiatry from 2004 [14]. Its purpose is to train a core group of mental health professionals in case management and the use of individual service plans, to enable them to deliver training programs and to establish community-based services [14,15]. However, it is primarily for the patients with severe mental disorders, rather than those at the hospital outpatient department, so ensuring uniformity in standards of care and types of mental health services available across the country is a further challenge the government must deal with. In addition, one of the major goals of China’s new mental health law, which was adopted by the Standing Committee of the National People’s Congress on October 26, 2012, and took effect on May 1, 2013 [44], is to expand access to mental health services by shifting the focus of services from specialized psychiatric hospitals in urban centers to general hospitals and community health clinics in both urban and rural communities [45,46]. Many difficulties that block the achievement of this goal, including insufficient health providers in rural areas, limited training of community-based medical personnel, resistance of specialists who are unwilling to move services from hospitals to community settings, urban patients’ preference for treatment at hospital outpatient departments, insufficient necessary drugs at local clinics, poor coordination between inpatient and outpatient services, and the highly mobile population, will continue to exist in future [45]. The Chinese government is acutely aware of these issues and trying to solve them, including testing alternative mechanism [47] and refining the good experience and deficiency from previous pilot models (e.g. “686” model).

## Conclusion

This is the first pathway study in urban China. We discovered that patients first seek help from various sources prior to reaching a MHP due to lack of awareness of treatment services and fear of the stigma associated with mental disorders.

The study underlined the importance of improving skills and knowledge that will facilitate the recognition of psychiatric disorders in community health centers, the general hospital system, and by private practitioners. The pathway described here should be considered while preparing mental health programs in the future.

### Limitations

Our study has some limitations. First, this study was performed in the military general hospital, which is open to civilian patients from all over the country, the small sample size makes it difficult to evaluate the effect of variation in diagnostic categories and characteristics of participating facilities in China, although the patients in our study were defined as general population. Second, the convenience sampling was used in this study, so there is a potential selection bias on sampling patients. Third, information gathered in this study is based on the willingness of patients to acknowledge their previous sources of care. Thus, patients may have been reluctant to disclose contacts with cares, such as religious or traditional healers. Four, the reliability test on the accuracy of data by different psychiatrists, which was not performed, may affect the findings.

Despite these limitations, this study is noteworthy in that this is the first study on pathways to care for patients with mental health problems in Northern urban China, and this will shed light on the planning of psychiatric services in the region in the future. We hope that this study will generate hypotheses and studies focused on ways of improving the mental health care system in China.

## Competing interests

The authors declare that they have no competing interests.

## Authors’ contributions

All authors participated in the preparation of the semi-structured questionnaire based on the encounter form developed in the WHO collaborative study. DHT, XLZ, and ZYQ participated in the design of this study. WJZ, XML, YL, XHW, HWX, YRZ, YFL and MQG collected the data and assisted data analyses. WJZ, XML, and YL analyzed the data and wrote the first draft of the paper, and XHW, HWX, MQG, and YFL checked the draft. DHT, XLZ, ZYQ, WJZ, and AJ conducted the further revision of the manuscript. All authors read and approved the final manuscript.
